# Ketoprofen Recognition and Sensing by Zn(II) Complexes with Fluorogenic Triamine Receptors

**DOI:** 10.3390/molecules30234556

**Published:** 2025-11-26

**Authors:** Yschtar Tecla Simonini Steiner, Liviana Mummolo, Rania Zartit, Massimo Innocenti, Marco Bonechi, Matteo Savastano, Luca Prodi, Andrea Bencini, Riccardo Chelli, Giammarco Maria Romano

**Affiliations:** 1 Dipartimento di Chimica “Ugo Schiff”, Università degli Studi di Firenze, Via della Lastruccia 3, Sesto Fiorentino, 50019 Firenze, Italyrannosha86@hotmail.com (R.Z.); marco.bonechi@unifi.it (M.B.);; 2 Dipartimento di Chimica “Giacomo Ciamician”, Università degli Studi di Bologna, Via Gobetti 85, 40129 Bologna, Italy; 3 Dipartimento di Scienze Umane e Promozione della Qualità della Vita, Università San Raffaele Roma, Via di Val Cannuta 247, 00166 Roma, Italy

**Keywords:** ketoprofen, anthracene, zinc complexes, fluorescent receptors, anion binding, supramolecular chemistry, NSAIDs detection, molecular dynamics simulations

## Abstract

Ligands L1 and L2 are composed, respectively, by a diethylenetriamine or a dipropylenetriamine moiety linked at their extremities to anthracene units through methylene bridges and form stable 1:1 complexes with Zn(II), in which the metal is coordinated by all three nitrogens of the ligands. Zn(II) binding by L1 leads to a marked enhancement of the fluorescence emission, thanks to the inhibition of the photoinduced electron transfer (PET) process from the benzylic amine groups of the triamine sub-unit to the excited fluorophore, which normally quenches the emission of fluorescent polyamine receptors. Conversely, the emission of L2 is somewhat quenched by Zn(II) binding likely due—as also indicated by ab initio calculations and molecular dynamics simulations—to the formation of cation π quenching contacts between the metal and the anthracene moieties that overcome the effects of PET inhibition. The Zn(II) complexes of both ligands are able to bind ketoprofen (KP) in its anionic form, thanks to the formation of COO^−^—Zn(II) coordinative bonds, to form [KPZnL]^+^ and [(KP)_2_ZnL] (L = L1 or L2) ternary adducts. While KP binding to [ZnL2]^2+^ enhances the fluorophore emission, coordination of KP to [ZnL1]^2+^ slightly reduces the anthracene emission, due, once again, to the formation in the L1 ternary complexes of marked cation π contacts.

## 1. Introduction

Non-steroidal anti-inflammatory drugs (NSAIDs) represent a worldwide largely used class of pharmaceuticals with powerful painkiller, antipyretic, and anti-inflammatory effects [1,2,3]. In consequence, they represent drugs with widespread use in human therapies and ubiquitous veterinary consumption [4,5], including intensive, often uncontrolled, use in farming [5,6,7]. As such, they are released in soils and surface waters, although not yet accompanied by efficient methodologies for removal, storage, and disposal of waste, leading to their increasing presence in aquatic environment [8,9]. So far, they are considered emerging pollutants, i.e., chemicals that are often poorly regulated and commonly monitored in the environment, with known or suspected adverse ecological and/or human health effects [10,11,12]. Therefore, there is an effective challenge for economically viable, highly sensitive, and rapid response sensors to be used for the protection of human/animal health and ecosystems. Optical sensors can match these requirements [13,14,15]. Recently, several nanostructured receptors have been developed for NSAIDs optical detection, mainly consisting in functionalized quantum dots (QDs) or carbon dots (CDs). Examples include near-infrared fluorescence sensors based on copper-doped CdS QDs and zinc oxide nanorods for ketoprofen (KP) sensing [16], CdSe/ZnS nanoparticles for chiral discrimination of enantiomeric isomers of NSAIDs [17], and CdTe QDs [18], a molecularly imprinted material based on CDs [19]. Nanostructured materials based molecularly imprinted polymers functionalized with rhodamine or fluorescein [20], Ag nanoparticle assemblies [21,22,23] or arrays of monolayered Au nanoparticles [24], and, finally, fluorescent hydrophilic polyurethane films [25] have also been developed for the detection of NSAIDs. Less attention has been paid to molecular optical sensors for NSAIDs. The first examples have been cinchonine-based receptors for enantiomeric recognition of NSAIDs [26] and arrays of calixpyrrole-based fluorescent hosts for NSAIDs detection [27] and a BINOL-containing macrocycle, able to optically signal NSAIDs in non-aqueous solvents [28]. More recently, open chain and macrocyclic systems containing *N,N*-bis(3-squaramidoylpropyl)dansylamide units were used to detect KP and naproxen in CH_3_CN/DMSO [29] and triamine receptors equipped with two anthracene units for KP binding and sensing [30].

Most NSAIDs share some common structural characteristics. In fact, they possess a carboxylic group, deprotonated at neutral pH in aqueous solution, linked to a hydrophobic portion. Their interaction with artificial receptors is driven by hydrogen bonding between the carboxylate groups and H-bonding donor sites of the guests, although hydrophobic interactions can also be involved in the formation of the adducts. On the other hand, carboxylate groups are also known for their ability to bind transition metal cations. Therefore, the use of metal complexes with fluorescent molecular receptors may represent an alternative pathway for the development of simple probes for these pharmaceuticals. On the other hand, to the best of our knowledge, only a single example of fluorescent metal complex for NSAIDs sensing has been reported involving a dinuclear Zn(II) complex with a biphenol–dipicolylamine based receptor capable of detecting ketoprofen and ibuprofen in aqueous media [31].

**Scheme 1:**
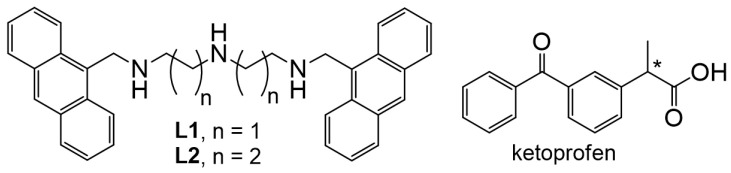
Fluorescent polyamines receptors (L1 and L2) and target analyte.

Receptors L1 and L2 (Scheme 1), containing, respectively, a diethylenetriamine or a dipropylenetriamine unit linked to an anthracene fluorophore, are able to detect KP at neutral pH in a water/ethanol (50:50 *v*/*v*) mixture through the formation of 1:1 and 1:2 complexes stabilized by electrostatic, hydrogen-bonding, and hydrophobic interactions, resulting in a marked fluorescence enhancement, especially for L1 [30]. However, L1 and L2 contain a triamine unit whose binding ability for transition metal is well known [32,33] and, therefore, the formation of stable Zn(II) complexes can be easily envisaged. At the same time, the metal coordination sphere is likely to be not fulfilled by the three amine donors of L1 and L2, making the Zn(II) ion an optimal anchoring point for the carboxylate group of NSAIDs. Finally, metal cations often change the emission properties of fluorescent ligands, and, in the case of Zn(II), the emission intensity is often enhanced by metal binding [34]. In light of these considerations, we decided to investigate the Zn(II) binding properties of L1 and L2 and the ability of the formed complexes to bind and optically detect KP. Our purpose is the comparison between the binding and sensing ability of the ‘free’ ligands and their Zn(II) complexes, taking KP as the example, one of the most common NSAIDs not featuring fluorescence emission, in order to elucidate the role of Zn(II) in NSAIDs optical signaling.

## 2. Results

### 2.1. Zn(II) Coordination with L1 and L2

**Figure 1:**
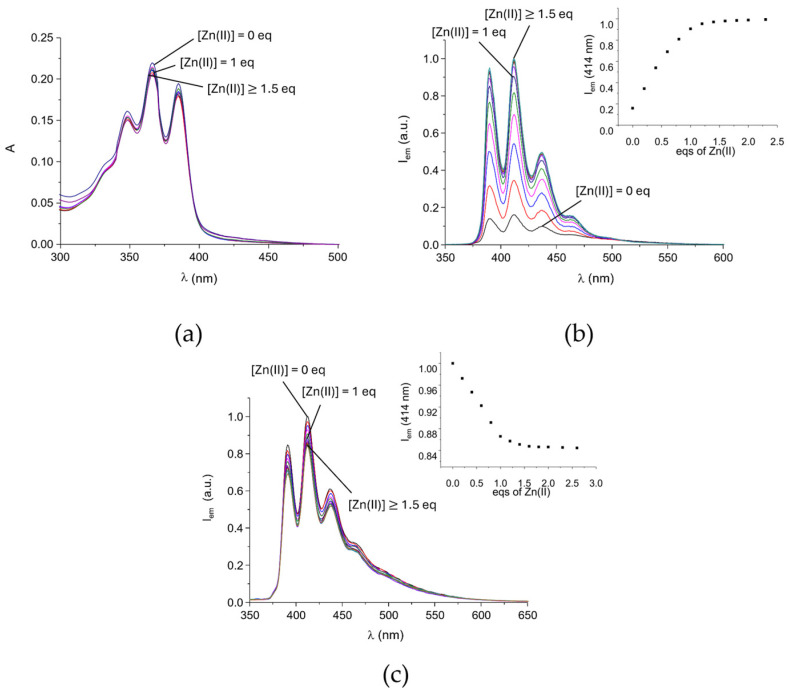
Absorption (**a**) and emission (**b**) spectra of L1 and emission spectra of L2 (**c**) in EtOH/H_2_O 50:50 (*v*/*v*) solution in the presence of increasing amount of Zn(II) at neutral pH value ([L1] = [L2] = 1 × 10^−5^ M, TRIS/HCl buffer 0.001 M, λ_exc_ = 340 nm, 298 K).

The analysis of Zn(II) coordination with L1 and L2 was first performed in EtOH/H_2_O 50:50 (*v*/*v*) solution by UV–Vis spectrophotometry and fluorescence emission spectroscopy. Ethanol was selected as the co-solvent to guarantee solubility of the Zn(II) complexes, without drastically altering the polarity of the medium. To this purpose, increasing amounts of Zn(II) were added to a 1 × 10^−5^ M solution of L1 or L2 in EtOH/H_2_O at pH 7 (TRIS buffer) and the results are shown in Figure 1 and Figure S1 (Supplementary Materials) for L1 and L2. As shown in Figure 1a and Figure S1 for L1 and L2, respectively, the absorption spectra of both ligands, featuring the characteristic absorption band of the anthracene chromophore, are almost not affected by the presence of Zn(II).

As previously reported [30], L1 is poorly emissive in EtOH/H_2_O at neutral pH value. At this pH, the receptor is mainly in its monoprotonated form [30], with the acidic proton being localized on the central nitrogen of the triamine unit. The terminal amine groups, adjacent to the anthracene moieties, can give an efficient photoinduced electron transfer (PET) process to the excited fluorophore [34], thus quenching its emission, as often observed in polyamine-based receptors containing appended fluorogenic units [34,35,36]. Addition of Zn(II) induces a marked increase in the typical emission band of anthracene centered at 414 nm (Figure 1b). As shown in the inset of Figure 1b, the emission intensity at 414 nm linearly increases up to a 1:0.9 ligand to metal molar ratio (R) to achieve a constant value for R > 1.5. In these conditions, the emission intensity undergoes a seven-fold increase with respect to the ‘free’ receptor at the same pH value, indicating the formation of a stable 1:1 Zn(II) complex, as expected considering the well-known ability of the 1,4,7-diethylenetriamine moiety to bind transition metals, including Zn(II), in aqueous media. Analysis of the spectral data with the HYPSPEC program [37] provides an apparent binding constant at pH 7.0 (ligand speciation not analytically considered) of 7.9(1) log units for the addition of Zn(II) to L1. A quantum yield (Φ) of 0.35 was found for the complex, which was much higher than that previously reported for the ligand (Φ = 0.083) at the same pH value [30]. The increasing emission with Zn(II) coordination is normally observed in polyamine-based fluorescent Zn(II) chelating agents and is generally ascribed to the involvement of the lone pairs of the amine groups in metal binding and to the consequent inhibition of the PET effect [34,35,36].

A different behavior is observed for the emission of L2 in the presence of the metal, as displayed in Figure 1c. In this case, the anthracene emission intensity undergoes a 15% decrease in the presence of a slight excess of the metal at pH 7.0 (Φ = 0.061 and 0.055 for the not-coordinated L2 ligand and its Zn(II) complex, respectively). Analysis of the spectral data with the HYPSPEC [37] program gives an apparent Zn(II) to L2 binding constant at pH 7.0 of 6.9(1) log units. The lower constant obtained for L2 matches well with the known ability of diethylenetriamine-based receptors to form more stable complexes with first-row transition metal cations with respect to dipropylenetriamine-containing receptors. This is due to the formation, in the former case, of penta-membered chelate rings, which are more stable than hexa-membered rings formed by the latter receptors [33,38,39]. This consideration could also explain the different effect of Zn(II) complexation on the fluorescent emission of the two triamine ligands. In fact, the Zn(II)-N bonds in the [ZnL2]^2+^ complex could be weaker than in [ZnL1]^2+^ and detachment, even partial, of a nitrogen donor atom could occur, leading to renewal of the PET effect that would justify the observed decrease in the emission upon Zn(II) coordination by L2. On the other hand, Zn(II) complexation is likely to also imply marked changes in ligand conformation that, in turn, could affect the emission properties of the anthracene moieties. From this point of view, the propylene chains linking the amine groups of L2 are more flexible than the ethylene ones in L1, and, therefore, different conformations of the two ligands in their Zn(II) complexes can be easily envisaged. In turn, this would imply changes in the mutual disposition of two anthracene units, with the formation of C-H π, π-π, or cation-π interactions and a different solvation of the anthracene units.

**Figure 2:**
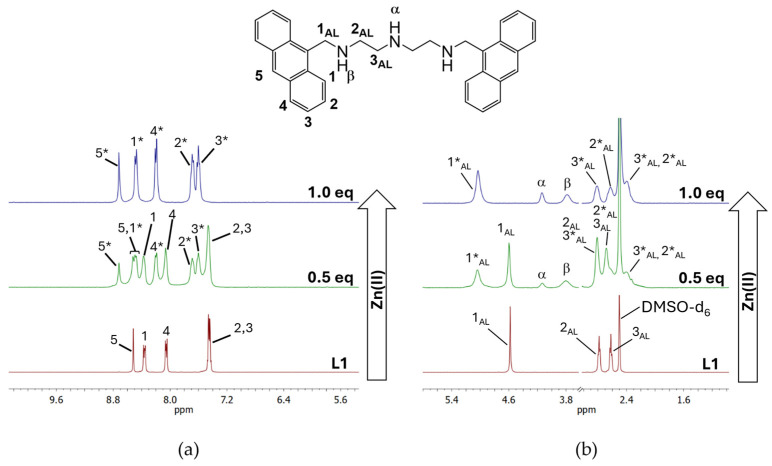
Aromatic (**a**) and aliphatic (**b**) portions of the ^1^H NMR spectrum of L1 in the absence and in the presence of 0.5 and 1 equivalent of Zn(II) in DMSO at 298 K ([L1] = 0.01 M). Starred numbers indicate the ^1^H signals of the Zn(II) complex, Greek letters indicate the resonances of the NH protons.

As a first attempt to analyze the structural changes in the L1 and L2 receptors upon Zn(II) complexation, we performed ^1^H NMR titrations by adding increasing amounts of Zn(II) to solutions of L1 and L2. The ligands and their Zn(II) complexes did not show sufficient solubility in EtOH/H_2_O (50:50, *v*/*v*) solution at pH 7.0 at the concentrations used for NMR experiments, preventing the ^1^H NMR analysis in this medium. Therefore, all measurements were performed in d_6_-DMSO, in which the solubility of the receptors and their Zn(II) complexes is large enough to allow for the ^1^H NMR titrations (5 × 10^−3^ M ligand concentration). Although DMSO is expected to exhibit stronger coordination toward Zn(II), the primary aim of this study is to rationalize the structural changes in the ligands upon metal coordination. Thus far, the use of DMSO as the solvent for NMR measurements appears appropriate. The aromatic and aliphatic portions of the ^1^H NMR spectra of L1 solutions with different amounts of Zn(II) are reported in Figure 2. We can clearly note that the addition of 0.5 equivalents of Zn(II) leads to the formation of a new sub-spectrum, overlapped to that of the free ligand L1, attributable to the formation in solution of a Zn(II) complex, slowly exchanging on the NMR time scale with the not Zn(II)-bound ligand. The addition of 1 equivalent of Zn(II) leads to the complete disappearance of the sub-spectrum of the free ligand (see Figure 2), suggesting the complete formation of the complex. In fact, although the presence of DMSO may reduce the stability of the Zn(II) complexes with the two ligands, due to stronger metal-solvent interactions, it does not significantly prevent their formation. The signals of the aliphatic chain of L1 in its Zn(II) complex appear fluxional and partially superimposed with the DMSO signal, and, therefore, they are poorly informative of the conformation of the aliphatic chain (Figure 2b). However, they point out the removal of the overall *C_2v_* symmetry, averaged on the NMR time scale. The complex assumes a *C_s_* symmetry, likely due to the increased rigidity of the ligand backbone upon metal complexation, as confirmed by the appearance in the aliphatic part of the spectrum (Figure 2b) of the signals of the NH protons, not observable in the free ligand spectrum, because of their fluxionality.

As shown in Figure 2a, the addition of Zn(II) induces a downfield shift in all the anthracene signals, the resonances of the 2, 3, and 5 protons being most affected by metal complexation (more than 0.2 ppm downfield shift). This effect is also observed to a lesser extent in the case of L2, although, in this case, fluxionality affects not only the aliphatic but also the aromatic region of the spectrum, and the shifts in the ^1^H signals are less affected by Zn(II) coordination (Figure S2). In particular, for L2, the resonance of proton 5 experiences a detectable downfield shift upon Zn(II) addition, while the remaining aromatic and aliphatic signals become broadened and more fluxional, showing only minor chemical shift variations. This is likely due to the larger flexibility of L2 propylenic chains with respect to the ethylenic ones of L1 that makes, on the NMR time scale, the exchange faster between the L2 and its Zn(II) complex. The downfield shifts observed for the aromatic protons upon Zn(II) coordination can be due to different effects, including the formation of stacking interactions between the anthracene units or cation-π interaction involving the charged metal center. Changes in the hydrophobic characteristics of the aromatic proton surroundings can also affect the ^1^H NMR chemical shifts. As anticipated, these effects can also affect the anthracene fluorescence emission properties.

They imply conformational changes upon Zn(II) coordination that, in turn, are related to the Zn(II) coordination environment. With this in mind, we performed ab initio calculations and molecular dynamics (MD) simulations to elucidate the conformational changes in L1 and L2 occurring upon metal binding.

**Figure 3:**
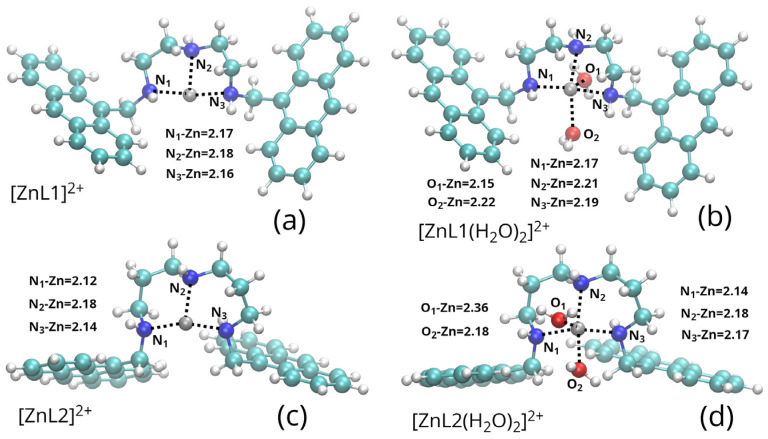
Ball and stick representations of energetically optimized structures of the [ZnL1]^2+^ (**a**), [ZnL1(H_2_O)_2_]^2+^ (**b**), [ZnL2]^2+^ (**c**) and [ZnL2(H_2_O)_2_]^2+^ (**d**) complexes, obtained from ab initio calculations. Cyan: C, blue: N, red: O, white: H, gray: Zn. The Zn-N and Zn-O bond distances (Å) are also shown.

Ab initio structural optimizations based on energy minimization have been performed to estimate the Zn-N bond distances in the metal complexes with L1 and L2 to be used in the semiempirical binding potential of the MD simulations. Furthermore, the analysis of the coordination sphere of the metal and the evaluation of the Zn-N bond distances can be informative of the different fluorescence emission observed for the complexes with L1 and L2, as the presence of weakly bound or unbound amine donors can quench the fluorescence of the anthracene moieties via PET processes to the excited fluorophore. In particular, a stronger binding is expected to yield a larger PET inhibition and, ultimately, a larger enhancement of the fluorescence emission. Noting that the strength of a Zn-N bond is associated with the respective bond distance, we would therefore expect shorter Zn-N bond distances in [ZnL1]^2+^ with respect to [ZnL2]^2+^. We performed ab initio calculations on both the [ZnL]^2+^ and [ZnL(H_2_O)_2_]^2+^ complexes (L = L1 or L2). Zn(II), in fact, often presents penta-coordination, in which water molecules fulfill the coordination sphere of the metal. The energetically optimized structures are shown in Figure 3, where we have also reported the Zn-N and Zn-O (relative to coordinated water molecules) bond distances.

The Zn-N distances are very similar in the L1 and L2 complexes, ranging from 2.12 to 2.21 Å, and not significantly affected by the presence of coordinated water molecules. These results suggest that PET inhibition may comparably affect [ZnL1]^2+^ and [ZnL2]^2+^, ruling out its main role in modulating the differences between the fluorescence emission intensity of the two complexes.

**Figure 4:**
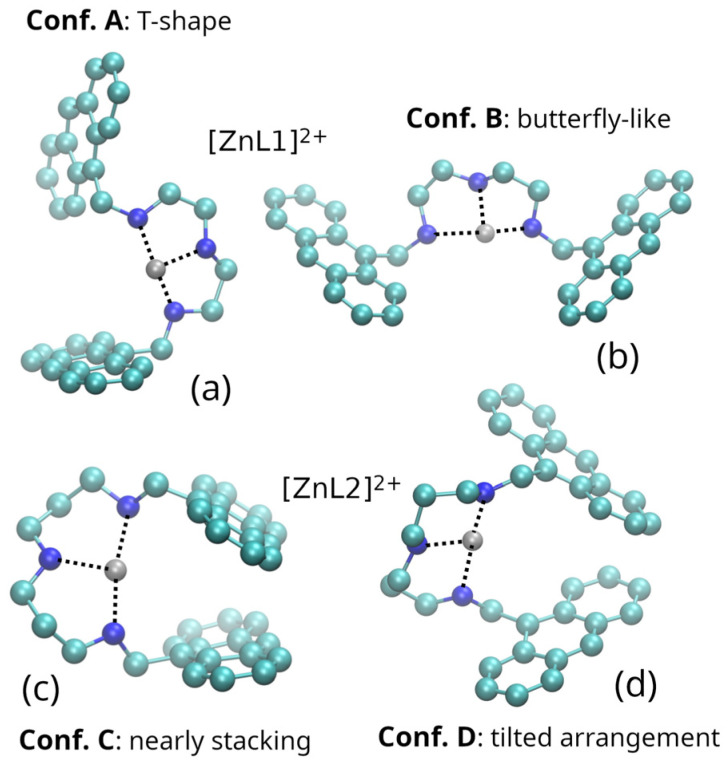
Representative structures in ball and stick style of the conformers observed in the MD simulations of [ZnL1]^2+^ (Conf. A (**a**) and Conf. B, (**b**)) and [ZnL2]^2+^ (Conf. C (**c**) and Conf. D (**d**)). Cyan: C, blue: N, gray: Zn. The H atoms and solvent molecules, including metal bound water molecules, are not displayed for the sake of clarity.

More interestingly, the MD simulations performed on the [ZnL1]^2+^ and [ZnL2]^2+^ complexes in the EtOH/H_2_O (50:50, *v*/*v*) solution point out that the conformations assumed by the two ligands in their complexes show remarkable differences (Figure 4).

**Figure 5:**
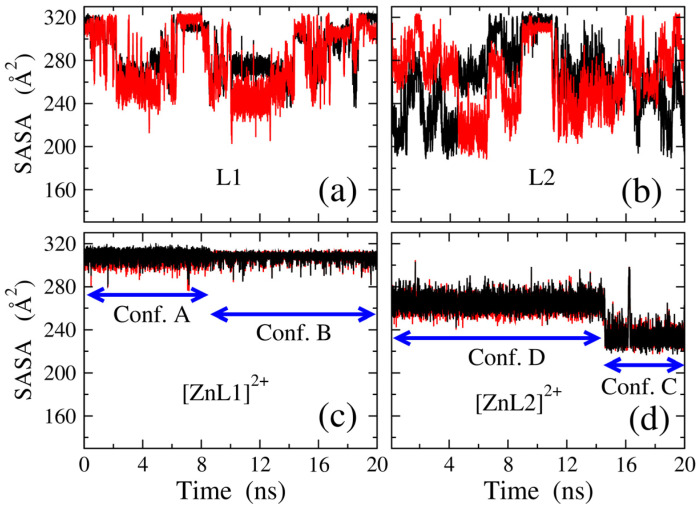
SASA in Å^2^ of the two anthracene moieties (black and red lines) as a function of time (ns) for the species L1 (**a**), L2 (**b**), [ZnL1]^2+^ (**c**), and [ZnL2]^2+^ (**d**). The time ranges in which conformers Conf. A, Conf. B, Conf. C, and Conf. D are observed (see Figure 4) are reported as double-side arrows in the panels (**c**,**d**).

In fact, the [ZnL1]^2+^ complex is characterized by two radically different conformations, in which the anthracene moieties are in turn arranged in T-shaped and butterfly-like arrangements (Conf. A and Conf. B in Figure 4a and Figure 4b, respectively). Instead, the [ZnL2]^2+^ complex displays more stacked arrangements (Conf. C and Conf. D in Figure 4c and Figure 4d, respectively), especially in the Conf. C conformation. The preferential stacked arrangement in the [ZnL2]^2+^ complex may ultimately result from the increased flexibility provided by the propylene chains compared to the ethylene chains in [ZnL1]^2+^. This enhanced flexibility can reduce steric interactions within the complex, facilitating structural rearrangements that favor a stacked disposition within an overall more compact ligand conformation. The flexibility generated by the propylene linkers can also be correlated to another aspect observed in the simulations, namely the increase in the average distance between the two distal N atoms (specifically those closest to the anthracene moieties) in the [ZnL2]^2+^ complex with respect to [ZnL1]^2+^ (4.30 Å against 4.05 Å). The different conformations assumed by the [ZnL1]^2+^ and [ZnL2]^2+^ complexes can be characterized by different intramolecular interactions involving the anthracene units and hence can play a role in tuning their solvation, that, in turn, can influence their fluorescence emission properties. In order to evaluate semi-quantitatively the role of solvation of the anthracene moieties in quenching processes (solvent is known to play a significant role in modulating quenching of aromatic systems), we have estimated the amount of surface area of the anthracene units exposed to the solvent. This estimate has been performed by computing the so-called solvent accessible surface area [40,41] (SASA) of the emitting anthracene units. The SASA calculation has been carried out using the algorithm by Lee and Richards [40] through the FreeSASA open-source C library for SASA calculations [41]. In general, the SASA calculation quantifies the overall interactions between solute and solvent (anthracene units and water/ethanol mixture in our case) in an average way, namely disregarding specific structural arrangements. In Figure 5, we report the SASA of the two anthracene units as a function of time for the complexes under study. In general, the time behavior of the SASA reveals fast fluctuations due to high frequency vibrational motions (translational and librational vibrations of the molecules, as well as intramolecular modes) overlapped to slower but larger fluctuations arising from relevant structural rearrangements (this is particularly evident in Figure 5a,b). These motions are fast enough to allow the SASA fluctuations shown in Figure 5 to give qualitative, but significant, insights into the structural features of the observed conformers for the investigated metal complexes. From the time dependence of the SASA, we infer that the formation of the ${\left[\mathrm{ZnL}1\right]}^{2+}$ and ${\left[\mathrm{ZnL}2\right]}^{2+}$ complexes leads to a significant decrease in the flexibility of the L1 and L2 scaffolds. The systems pass from a dynamical situation featured by large and frequent conformational changes (Figure 5a,b) to almost conformationally frozen dynamics (Figure 5c,d). Correspondingly, we observe a decrease in the number of conformational states in the [ZnL1]^2+^ and [ZnL2]^2+^ complexes with respect to the free receptors. In particular, almost all molecular conformations observed in the simulations of these complexes can be classified in two structural motifs for each complex, whose representative arrangements are displayed in Figure 4. In Figure 5c,d, we mark the simulation time ranges in which each conformer is observed. As anticipated, the [ZnL1]^2+^ complex is characterized by two remarkably different conformations, in which the anthracene moieties are arranged in T-shaped and butterfly-like shapes, while the [ZnL2]^2+^ complex displays more stacked dispositions, especially in the Conf. C arrangement (Figure 4). This conformation is responsible for the significant decrease in the SASA in the last part of the simulation of the [ZnL2]^2+^ complex (see Figure 5d).

In order to obtain a semi-quantitative, but synthetic, view of the system solvation, we have computed the time averaged SASA of the anthracene units (Table S1). Overall, no significant differences are observed between the L1 and L2 free ligands and the corresponding Zn complexes. These outcomes poorly correlate with the emission changes upon Zn(II) binding (Figure 1), suggesting that solvation does not play a determinant role in modulating the fluorescence emission.

**Figure 6:**
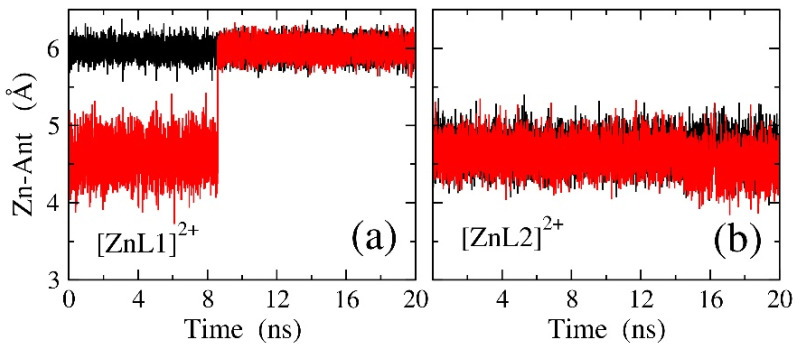
Distances (in Å) between the Zn(II) cation and the centers of mass of the two anthracene moieties (black and red lines) as a function of time (in ns) for the complexes [ZnL1]^2+^ (**a**) and [ZnL2]^2+^ (**b**).

More interestingly, the analysis of the distances between the metal cation and the centers of mass of the anthracene moieties as a function of time (Figure 6a,b) reveals a radically different behavior of L1 and L2. In fact, while the distance is as small as 4.7 Å, or less, in [ZnL2]^2+^, consistent with a significant interaction between Zn^2+^ and both anthracene units, in [ZnL1]^2+^ it takes much larger values (*ca* 6 Å) for both units, even if in the first part of the simulation the distance of one anthracene unit from the metal cation lies at about 4.6 Å. Considering that cation-π interactions are known to quench the emission of excited fluorophores [42,43], the fact that Zn^2+^ coordination with L2 slightly reduces the emission of the anthracene fluorophore may be attributed to the formation of Zn^2+^ π contacts, as observed in MD simulations. In the case of the L1 complex, these interactions are likely inhibited by the more rigid structure of the ligand that would prevent the aromatic units to assume a spatial disposition close to the metal center. From the structural standpoint, the trend of the Zn-anthracene distance of Figure 6a,b confirms the presence of two different conformations for both [ZnL]^2+^ complexes (the conformations reported in Figure 4), as already noted in discussing the SASA (Figure 5c,d). We point out that, in the simulation time one conformational jump is actually observed in both [ZnL1]^2+^ and [ZnL2]^2+^ complexes at about 8 and 14 ns, respectively, which is evident in Figure 6a,b as well as in Figure 5c,d.

### 2.2. Ketoprofen Binding by the Zn(II) Complexes with L1 and L2

**Figure 7:**
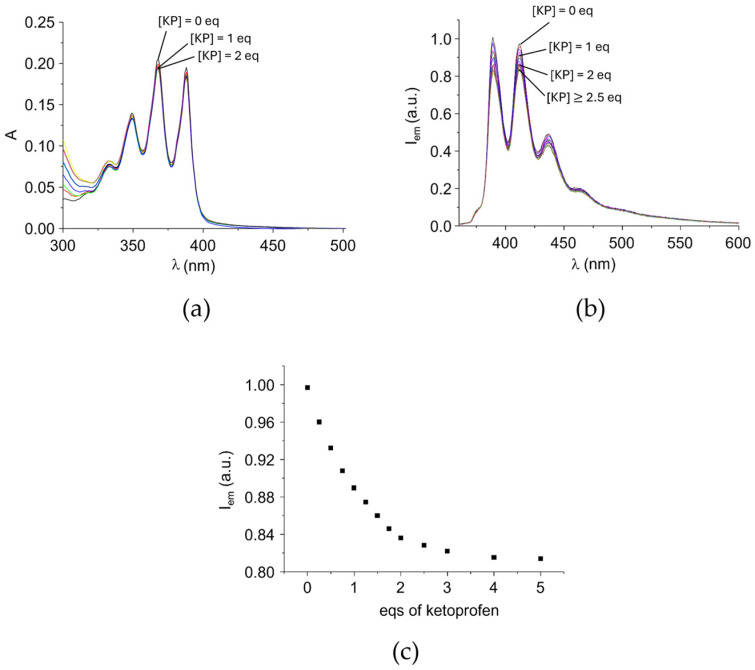
(**a**) Absorption and (**b**) emission spectra of [ZnL1]^2+^ at pH 7.0 in EtOH/H_2_O 50:50 (*v*/*v*) solution in the presence of increasing amounts of KP ([[ZnL1]^2+^] = 1 × 10^−5^ M, TRIS/HCl buffer 0.001 M, λ_exc_ = 340 nm, 298 K). (**c**) Plot of the fluorescence emission of L1 at 414 nm.

**Figure 8:**
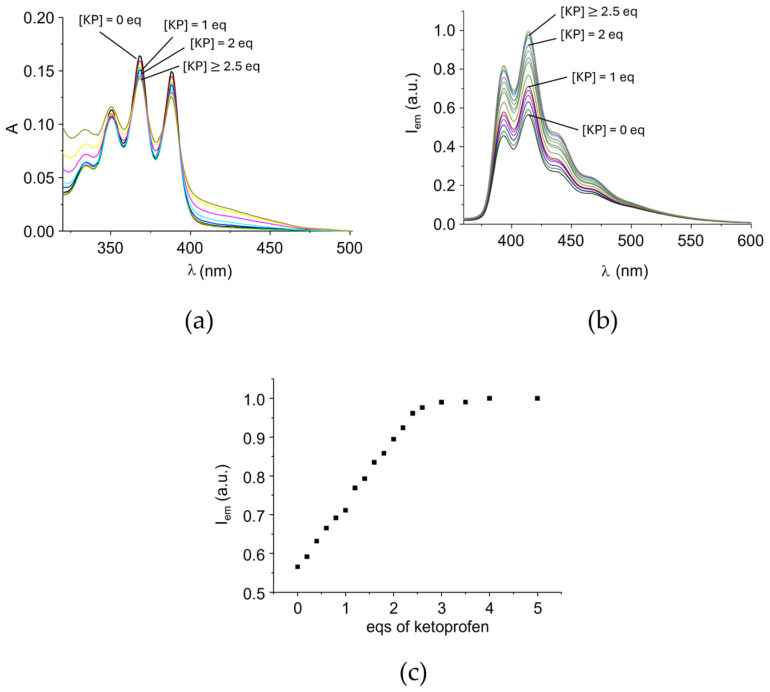
(**a**) Absorption and (**b**) emission spectra of [ZnL2]^2+^ at pH 7.0 in EtOH/H_2_O 50:50 (*v*/*v*) in the presence of increasing amounts of KP ([ZnL2]^2+^ = 1 × 10^−5^ M, TRIS/HCl buffer 0.001 M, λ_exc_ = 340 nm, 298 K); (**c**) plot of the fluorescence emission of L2 at 414 nm.

As shown by the results outlined above, in the Zn(II) complexes with L1 and L2, the coordination sphere of the metal center is not saturated by the ligand donors. The metal is likely to complete its coordination environment via binding of water molecules, which can be replaced by donor group(s) from exogenous substrates, such as KP. Of note, KP is in anionic form at pH 7.0 [30] and, therefore, possesses a carboxylate group suitable for Zn(II) binding. In the light of these considerations, both the L1 and L2 complexes with Zn(II) can be exploited as potential metal-based receptors for this NSAID. To this purpose, we performed UV–Vis and fluorescence emission measurements, by adding increasing amounts of KP to a 50:50 *v*/*v* EtOH/H_2_O solution, buffered at pH 7.0, of each Zn(II) complex. As in the case of the receptors in the absence of Zn(II), the absorption spectra do not show relevant changes upon the addition of the substrate (Figure 7a and Figure 8a). Conversely, the fluorescence emission of both [ZnL1]^2+^ and [ZnL2]^2+^ is affected by the interaction with KP. The addition of KP to a solution of the [ZnL]^2+^ complex (L = L1 or L2) induces a slight decrease in the anthracene emission in the case of L1 (Figure 7b,c), while a marked enhancement of the intensity is observed in the case of L2 (Figure 8b,c), that is the opposite behavior observed for Zn(II) binding by L1 and L2. In particular, the emission of [ZnL2]^2+^ increases almost linearly up to the addition of 2 equivalents of KP, to reach an almost constant value for a KP: [ZnL2]^2+^ molar ratio slightly greater than two. This feature suggests the formation of 1:2 adducts between the binary Zn(II) complexes and KP, in good agreement with the ability of Zn(II), which is coordinated by the three nitrogens of the receptors in the metal complexes, to easily expand its coordination sphere, achieving an overall penta-coordination. Compared to [ZnL]^2+^ complexes (L = L1 or L2), the emission at 414 nm is almost two-fold enhanced in the case of L2 and *ca* 18% reduced in the case of L1 in the presence of 4 equivalents of KP, with quantum yields Φ of 0.10 and 0.28 for the ternary complexes formed by L2 and L1, respectively, (Φ= 0.06 and 0.35 for the [ZnL2]^2+^ and [ZnL1]^2+^ binary complexes, respectively).

Analysis of the spectral data with the HYPSPEC program leads to estimate apparent binding constants at pH 7.0 for the successive additions of one KP anion to [ZnL1]^2+^ (log K_1_ = 5.1(1)) and to its [KPZnL1]^+^ complex (log K_2_ = 4.0(2)). Although the spectral variations observed beyond 1 equivalent of added KP are small, they remain significant and reasonably indicate the formation of a minor 1:2 species. Despite the relatively low signal-to-noise ratio in this region, the confidence for a model that also considers a 1:2 species is quite similar to that found for a model that takes into account only a 1:1 complex. However, in this latter case, the log K value for the 1:1 species appears to be relatively high due to the omission of the 1:2 species from the model. Indeed, a model that considers the formation of both 1:1 and 1:2 species fits the titrations curve. In the case of the [ZnL2]^2+^ complex, data analysis does not allow for the calculation of a binding constant for each addition step, but only for an overall addition constant of 10.4(1) log units for the equilibrium [ZnL2]^2+^ + 2 KP = [(KP)_2_ZnL2]. This can be due either to the simultaneous addition of two KP anions to the complex or, more likely, to successive binding to the complex of single KP anions with similar addition constants, to form first a species and then the [(KP)_2_ZnL2] complex. The formation of both 1:1 and 1:2 complexes between the Zn(II)-based receptors and KP resembles the behavior of the metal-free L1 and L2 ligands. The constants found for the addition of KP to both Zn(II) complexes are similar or slightly lower than those measured for the corresponding metal-free analogs in their protonated form, determined by spectrofluorimetric titrations in the same experimental conditions (log K = 5.7 and 5.5 for the addition of a single KP anion to L1 and its Zn(II) complex, respectively, and 5.4 and 4.9 for the addition of a single KP anion to L2 and to its metal complex, respectively) [30]. The metal cation acts as anchoring point for the KP anions, forming coordinative bonds normally stronger than salt bridge interactions. However, in the absence of the metal, the binary complexes are stabilized not only by the NH_2_^+^–OOC– salt bridges, but also by hydrophobic interactions and hydrogen bonding contacts between the carbonyl group of KP and an amine group of the receptor, as shown by the crystal structure of the host–guest adduct [30]. The multiple interactions formed by anionic KP with the metal-free protonated receptor may explain the similar constants found for the addition of KP to L1 or L2 with respect to those found for KP binding by the binary Zn(II) complexes with L1 or L2.

**Figure 9:**
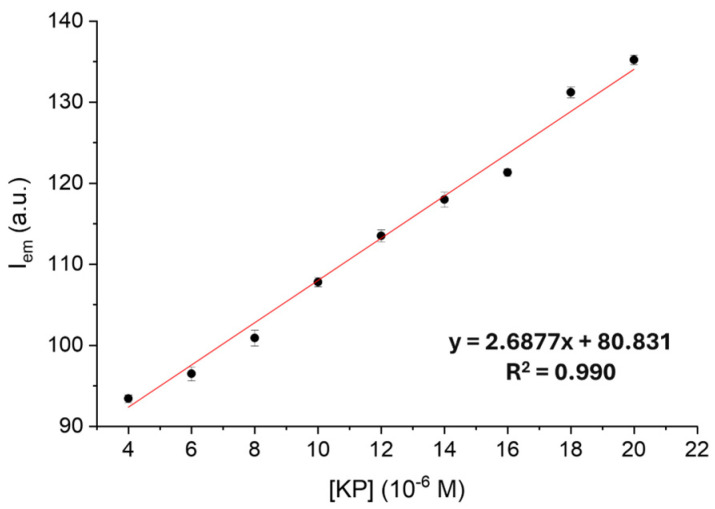
Plot of the fluorescence emission at 414 nm of [ZnL2]^2+^ ([[ZnL2]^2+^] = 1 × 10^−5^ M, TRIS/HCl buffer 0.001 M, λ_exc_ = 340 nm, σ = 0.495) at pH 7.0 in EtOH/H_2_O 50:50 (*v*/*v*) solution in the presence of increasing amount of KP at 298 K.

A 0.55 µM LOD value for the [ZnL2]^2+^ complex was estimated in EtOH/H_2_O 50:50 (*v*/*v*) solution at pH 7.0, by using the linear fit reported in Figure 9, while in the case of the [ZnL1]^2+^ complex the observed slight change in emission upon KP binding prevents a reliable calculation. To note, the LOD value found for [ZnL2]^2+^ is close to that measured in absence of the metal (0.28 µM).

Despite the different binding ability of the two Zn(II) complexes for KP, the most interesting finding is the observed optical response to KP binding displayed by the two complexes.

While KP coordination by the Zn(II) complex with L2 induces a marked increase in the emission, the formation of the ternary complex decreases the emission of L1, a markedly different behavior with respect to that found for the metal-free receptors, in which KP binding at pH 7.0 in EtOH/H_2_O 50:50 (*v*/*v*) solution induces a great enhancement of the L1 emission and a minor increase in the case of L2. The observed behavior—the [ZnL1]^2+^ and [ZnL2]^2+^ emissions are quenched and enhanced in the presence of KP, respectively—cannot simply be due to a different binding affinity of the two complexes for KP, but is likely related to conformational changes, induced by KP anchoring, that modulate the optical response.

Unfortunately, the ternary complexes suffer from poor solubility in EtOH/H_2_O and DMSO mixtures at the concentration employed in ^1^H NMR experiments, preventing their study by using this technique. Therefore, as in the case of Zn(II) binding by L1 or L2, we also performed ab initio calculations on the KP-containing ternary complexes, [KPZnL]^+^ and [(KP)_2_ZnL], taking into account the possible formation of a [KPZnL(H_2_O)]^+^ complex from [KPZnL]^+^, in which a water molecule allows Zn(II) to achieve penta-coordination. Based on the experimental results, it was considered useful and informative to consider both the 1:1 and 1:2 stoichiometries for the calculations. The ab initio optimized structures, as well as the Z-N bond distances, are displayed in Figure S3. Also in these complexes, the Z-N distances do not significantly differ from one another and neither do they differ from those found in the KP-free complexes of Figure 3. Therefore, ab initio calculations allow us to exclude a determinant role of the PET inhibition in the spectral differences in these compounds.

**Figure 10:**
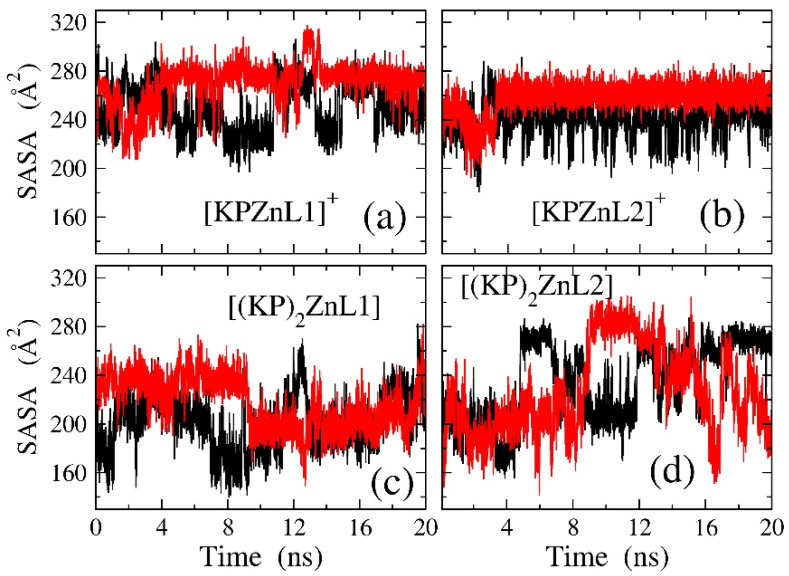
SASA (in ${Å}^{2}$) of the two anthracene moieties (black and red lines) as a function of time (in ns) for the complexes [KPZnL1]^+^ (**a**), [KPZnL2]^+^ (**b**), [(KP)_2_ZnL1] (**c**), and [(KP)_2_ZnL2] (**d**).

MD simulations allowed us to evaluate the SASA of the aromatic units, whose emission is generally affected by solvation (Figure 10). In the cases of the complexes with one and two KP molecules, a significant increase in the low-rate SASA fluctuations with respect to the KP-free complexes is observed (compare Figure 10 with Figure 5c,d).

**Figure 11:**
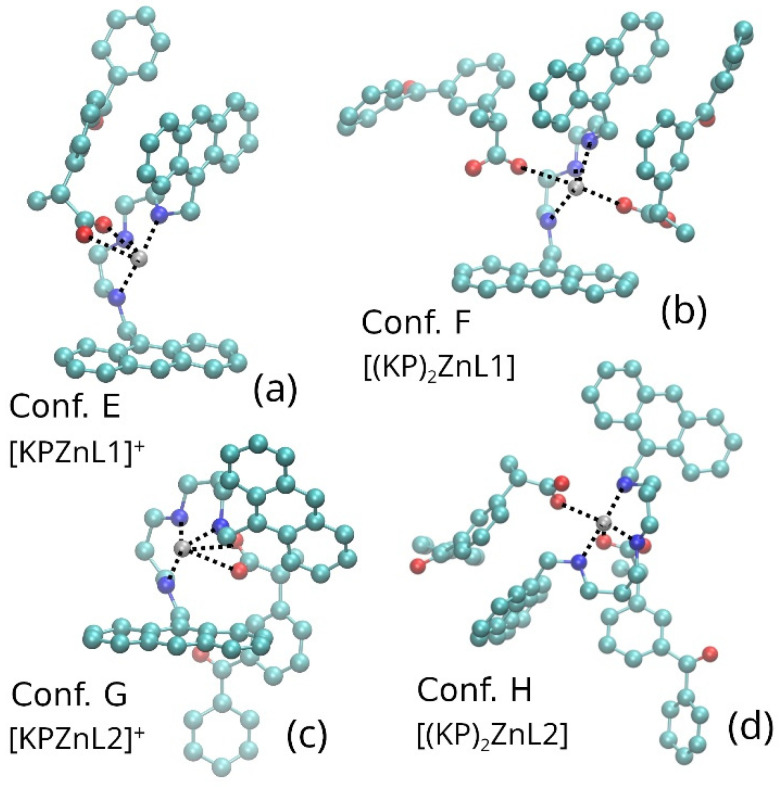
Representative structures in ball and stick style of the conformers observed in the MD simulations of [KPZnL1]^+^ (Conf. E) (**a**), [(KP)_2_ZnL1] (Conf. F) (**b**), [KPZnL2]^+^(Conf. G) (**c**), and [(KP)_2_ZnL2] (Conf. H) (**d**). Cyan: C, blue: N, red: O, gray: Zn. The H atoms and the solvent molecules are not displayed for the sake of clarity.

Of note, only one main structural motif has been revealed for each of the four complexes [KPZnL]^+^ and [(KP)_2_ZnL] (L = L1 or L2), whose representative structures are reported in Figure 11.

However, it is worth noting that the bounded KP molecules are characterized by some degree of flexibility. This residual flexibility is responsible for the large fluctuations of the SASA of the anthracene moieties (see Figure 10). In other terms, the SASA fluctuations in the KP-complexes are yielded by continuous rearrangements of the KP molecules, sometime interacting with the anthracene units (low values of the SASA), sometime moving far from them (large values of the SASA).

In summary, concerning the structural properties of the L1 and L2 scaffolds, we notice that for the L1 receptor, while complexation with $Zn^{2+}$ leads basically to two types of conformations (Conf. A and Conf. B in Figure 4), the addition of KP gives rise to nearly T-shaped conformations pretty similar to Conf. A, namely Conf. E and Conf. F in the [KPZnL1]^+^ and [(KP)_2_ZnL1]^+^ complexes, respectively (Figure 11). In the case of L2, the changes upon complexation with Zn(II) and KP are more radical, going from stacked-like conformations in the [ZnL2]^2+^ complex (Conf. C and Conf. D in Figure 4), to an open conformation in the [(KP)_2_ZnL2] complex (Conf. H, Figure 10d), passing through a tilted conformation in the [KPZnL2]^+^ complex (Conf. G, Figure 10c).

**Figure 12:**
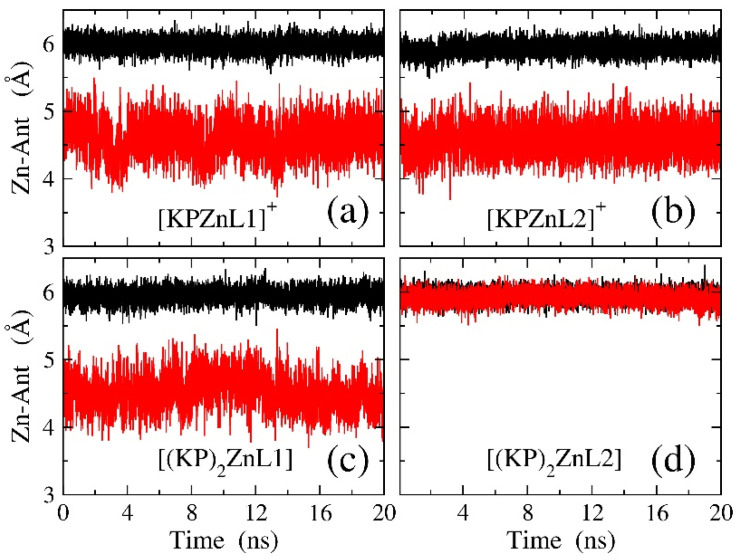
Distances (in Å) between Zn(II) and the centers of mass of the two anthracene moieties (black and red lines) as a function of time (in ns) for the species [KPZnL1]^2+^ (**a**), [KPZnL2]^+^ (**b**), [(KP)_2_ZnL1] (**c**), and [(KP)_2_ZnL2] (**d**).

The time evolution of the Zn-anthracene distance in the complexes formed with KP is shown in Figure 12. In the complex [KPZnL1]^+^ (Figure 12a), the anthracene units show a different behavior, one being close to Zn(II) (ca 4.6 Å), with the other being as far as 6 Å from the cation. Comparing this trend with that observed for [ZnL1]^2+^ (Figure 6a), we may infer that KP binding produces a global increase in the Zn-anthracene interactions and ultimately the occurrence of an enhanced quenching, as observed in the emission spectra (Figure 7), where quenching leads to a decrease in the fluorescence emission while increasing the amount of KP. An opposite change in the distances is observed for the [KPZnL2]^+^ complex (Figure 12b) compared to [ZnL2]^2+^ (Figure 6b). In the complex with KP, we observe conformations where one anthracene unit is far from the Zn^2+^ cation (at about 6 Å), while the other remains close to it (at about 4.5 Å). This would imply a decrease in the quenching effect due to the formation of a single Zn^2+^ π contact in the [KPZnL2]^+^ with respect to the [ZnL2]^2+^ complex, in which both anthracene units are close to Zn^2+^ (see Figure 6b), justifying the increase in the emission of the KP ternary complex [KPZnL2]^+^ with respect to [ZnL2]^2+^ one (see Figure 1c and Figure 8c). Similarly, in the [(KP)_2_ZnL2] complex (Figure 12d), both anthracene moieties are far from the metal cation (6 Å), in keeping, once again, with the fluorescence emission increase upon KP binding.

## 3. Materials and Methods

Ligands L1 and L2 were synthetized as previously reported [30]. All reagents and solvents were purchased from Sigma-Aldrich Chemie GmbH (Kappelweg 1, 91625 Schnelldorf, Germany).

### 3.1. Synthesis of [(ZnL1)(ClO_4_)_2_]·2 H_2_O

A solution of Zn(ClO_4_)_2_·6 H_2_O (10.5 mg, 0.028 mmol) in MeOH (1 mL) was added to a solution of L1 (19.1 mg, 0.028 mmol) in MeOH (5 mL). BuOH (1 mL) was added to the solution, and the solvent was allowed to slowly evaporate. The obtained crystals were filtered and washed with BuOH. (13.2 mg, yield 63%). Anal. calcd. for C_34_H_37_Cl_2_N_3_O_10_Zn: C 52.09, H 4.76, N 5.36, Zn 8.34. Found: C 52.67, H 4.13, N 5.98, Zn 8.22.

### 3.2. Synthesis of [(ZnL2)(ClO_4_)_2_]·2 H_2_O

A solution of Zn(ClO_4_)_2_·6 H_2_O (11.2 mg, 0.030 mmol) in MeOH (1 mL) was added to a solution of L2 (18.1 mg, 0.030 mmol) in MeOH (5 mL). BuOH (1 mL) was added to the solution, and the solvent was allowed to slowly evaporate. The crystals obtained were filtered and washed with BuOH. (13.5 mg, yield 61%). Anal. calcd. for C_36_H_41_Cl_2_N_3_O_10_Zn: C 53.25, H 5.09, N 5.17, Zn 8.05. Found: C 53.70, H 4.89, N 5.46, Zn 8.14.

### 3.3. Electronic Absorption and Fluorescence Measurements

Absorption and fluorescecence emission spectra were recorded on a Jasco V-670 spectrophotometer and on a Horiba Scientific Fluoromax Plus spectrofluorimeter, respectively. Spectrophotometric and fluorimetric titrations were performed by using an already reported procedure [44,45]. For tritrations with metal, a 1 mM solution of ZnCl_2_ was added to 1 × 10^−5^ M solutions of the ligands, while for titrations with KP, a 1 mM solution of KP was added to 1 × 10^−5^ M solutions of the Zn(II) complexes of the two ligands. In emission spectra collection, an excitation wavelength of 340 nm was used. All solutions and measurements were performed in a water/ethanol 50:50 (*v*/*v*) mixture at pH 7.0, using TRIS/HCl buffer 0.001 M, at 298.0 ± 0.1 K. During titrations with Zn(II) and with KP, the absorbance values of the free ligand and the corresponding Zn(II) complexes at the excitation wavelength were below or equal to 0.1, indicating that inner-filter effects were negligible under the experimental conditions [34]. Therefore, no correction for inner-filter effects was applied to the emission spectra. In addition, KP does not exhibit any significant absorption bands at 340 nm, confirming that its contribution to excitation light attenuation is minimal. The luminescence quantum yields were determined using quinine sulfate in a 0.05 M H_2_SO_4_ aqueous solution (Φ = 0.53) as a standard reference [46].

### 3.4. NMR Measurements

NMR titrations were carried out by using a Bruker Avance 400 MHz instrument, in DMSO-d6 by the addition of zinc trifluoromethanesulfonate 0.2 M and KP sodium salt 0.1 M to 0.005 M solutions of L1 or L2, at 298.0 ± 0.1 K.

### 3.5. Molecular Dynamic Simulations

MD simulations have been performed on the L, [ZnL]^2+^, [KPZnL]^+^, and [(KP)_2_ZnL] species (L = L1 or L2) in a EtOH/H_2_O 50:50 (*v*/*v*) solution. Specifically, simulation samples are made, in turn, of one solute molecule of those listed above, immersed within a solution of 300 ethanol and 976 water molecules. We note that, as the central N atom of the L1 and L2 molecules is in the ammonium form at neutral pH, MD simulations of these species were carried out taking this N atom in the ammonium form. The constant-pressure (1 atm), constant-temperature (298 K) thermodynamic ensemble is adopted with standard periodic boundary conditions applied to a cubic box. The temperature control is achieved using a Nosé–Hoover thermostat [47,48], while the pressure is kept constant by the Parrinello–Rahman method with uniform scaling of the simulation box volume [49]. A full-atom potential model was adopted. Intermolecular interactions are treated according to the Lennard-Jones potential together with the Coulomb potential between point net charges localized on the atoms. The intramolecular interactions include harmonic stretching, harmonic bending, proper torsions, improper torsions, and, for atoms separated by more than two covalent bonds, Coulomb and Lennard-Jones interactions. Moreover, the Coulomb and Lennard-Jones interactions between atoms connected by three covalent bonds are scaled by the standard AMBER fudge factors (0.8333 and 0.5 for the Coulomb and Lennard-Jones pair interactions, respectively). As prescribed by the AMBER protocol, the cross-interactions for the Lennard-Jones terms were calculated using the Lorentz–Berthelot mixing rules. The AMBER-like ff99sb force field [50] in combination with atomic net charges computed through a RESP fit [51] at the HF/6–31G* level of theory was used to model the molecules forming the solute, namely L1, L2, and KP, while all pair interactions of the Zn^2+^ cation, apart from the Zn-N bonds, are computed according to the potential of Ref. [52]. The Zn-N bond was modeled through a stretching harmonic potential with a force constant of 70 kcal ${\mathrm{mol}}^{-1}$ ${Å}^{-2}$ and a bond distance of 2.16 Å for all complexes. This distance corresponds to the average value of the Zn-N bond distances computed by ab initio calculations, while the force constant has been chosen to obtain a frequency of an isolated Zn-N stretching mode of the order of 250 ${\mathrm{cm}}^{-1}$. The TIP3P model [53] has been adopted for water, while for ethanol we have used the potential of Ref. [54]. Constraints are enforced to covalent bonds involving H atoms. Coulomb forces are treated by the smooth particle mesh Ewald method [55] using a fourth order B-spline interpolation polynomial for the charges, an Ewald parameter of 0.43 ${Å}^{-1}$, and a grid spacing smaller than 1 Å for the fast Fourier transform calculation of the charge weighted structure factor. The cutoff distance for the non-bonding interactions is 12 Å. A five-time-step r-RESPA integrator [56] is employed for integrating the equations of motion, with the largest time step of 9 fs. Simulations lasting 20 ns have been carried out for each system. MD simulations have been performed using the ORAC program [57].

### 3.6. Ab Initio Calculations

Quantum mechanical calculations have been carried out using density functional theory, with the B3LYP functional [58,59,60] for the exchange–correlation energy and the D3 version of Grimme’s dispersion with Becke–Johnson damping [61] to account for the dispersion interactions. We have used the lanl2dz basis set [62] for Zn and the 6–31++G(d,p) basis set for all other atoms. Solvent (water) has been accounted for through the polarizable continuum model [63]. Energy-based geometrical optimizations have been performed on several systems: L, [ZnL]^2+^, [ZnL(H_2_O)_2_]^2+^, [KPZnL]^+^, [KPZnL(H_2_O)]^+^, and [(KP)_2_ZnL] (L = L1 or L2). In all calculations, the energy minimization procedure has been verified through a normal mode analysis to exclude the presence of imaginary frequencies. All ab initio calculations have been performed using the g16 suite of programs [64].

## 4. Conclusions

This work shows that fluorescence emission of both cation and anion complexes with polyamine-based receptors in aqueous media is not only determined by proton and electron transfer processes involving the amine groups, but also by subtle conformational changes occurring upon complex formation. The latter contributes to, and, in some cases, determines the emission properties of the fluorophore via the formation of weak, but relevant, intramolecular interactions. As previously reported, KP coordination to metal-free ligands L1 and L2 leads to marked fluorescence emission enhancements, at least for L1, determined by proton transfer processes between the amine/ammonium groups of the partially protonated ligands. In the Zn(II) complexes, the emission changes are subtly regulated by weak intramolecular interaction between the coordinated metal and the anthracene units, which, in turn, are tuned by KP binding to Zn(II). Zn(II) coordination to the triamine chain of L1 and L2 prevents protonation of the amine groups and, therefore, any proton transfer process between ammonium and amine groups. At the same time, metal binding inhibits PET processes from the lone pairs of the amine donors, in particular those adjacent to the anthracene units, which normally quench the excited fluorophores. Therefore, emission enhancement upon Zn(II) coordination would be expected. Nevertheless, this behavior is observed only in the case of the [ZnL1]^2+^ complex, while Zn(II) binding by L2 induces emission quenching. In fact, in [ZnL2]^2+^ the larger flexibility of the propylenic chains of L2 allows the coordinated metal to achieve a spatial disposition close to the anthracene units enabling the formation of Zn^2+^ π contacts that quenches the emission. This conformational change is less favored in [ZnL1]^2+^, where the Zn^2+^ π interactions appear weaker, and the fluorescence emission is mainly determined by PET inhibition. The opposite behavior is observed upon KP binding to Zn(II). In fact, in the case of [ZnL1]^2+^, the binding of KP favors the formation of stronger cation π contacts that tend to quench the complex emission, while in the case of [ZnL2]^2+^ coordination of KP favors more opened conformations, allowing the metal to stay at a larger distance from the fluorophore, thus enhancing the anthracene emission.

## Data Availability

The original contributions presented in this study are included in the article/Supplementary Materials. Further inquiries can be directed to the corresponding authors.

## Supplementary Materials

The following supporting information can be downloaded at: https://www.mdpi.com/article/10.3390/molecules30234556/s1: Table S1: Average SASA (${Å}^{2}$) of the anthracene units of the systems under study. For each system, the SASA has been computed averaging over the time and over the two anthracene moieties; Figure S1: Absorption spectra of L2 in the presence of increasing amount of Zn(II) at neutral pH values ([L2] = 1 × 10^−5^ M, TRIS buffer 0.001 M, λ_exc_ = 340 nm); Figure S2: Aliphatic (a) and aromatic (b) portions of the ^1^H NMR spectrum of L1 in the absence and in the presence of 0.5 and 1 equivalent of Zn(II) in DMSO at 298 K ([L1] = 0.01 M). Starred numbers indicate the ^1^H signals in the Zn(II) complex; Figure S3: Ball and stick representations of energetically optimized structures of the [KPZnL]^+^ (a,b), [KPZnL(H_2_O)]^+^ (c,d) and [(KP)_2_ZnL] complexes (with L = L1 or L2), obtained from ab initio calculations. Cyan: C, blue: N, red: O, white: H, gray: Zn. The Zn-N and Zn-O(KP) bond distances (Å) are also shown.

## Abbreviations

The following abbreviations are used in this manuscript:

PET	Photoinduced Electron Transfer
KP	Ketoprofen
NSAIDs	Non-Steroidal Anti-Inflammatory Drugs
QDs	Quantum Dots
CDs	Carbon Dots
EtOH	Ethanol
TRIS	Tris(hydroxymethyl)aminomethane
NMR	Nuclear Magnetic Resonance
MD	Molecular Dynamics
SASA	Solvent Accessible Surface Area
LOD	Limit Of Detection
MeOH	Methanol
BuOH	Butanol

## References

[1] Wongrakpanich S., Wongrakpanich A., Melhado K., Rangaswami J. (2018). A Comprehensive Review of Non-Steroidal Anti-Inflammatory Drug Use in The Elderly. Aging Dis..

[2] Jahnavi K., Pavani Reddy P., Vasudha B., Narender B. (2019). Non-Steroidal Anti-Inflammatory Drugs: An Overview. J. Drug Delivery Ther..

[3] Vishwakarma R.K., Negi D.S. (2020). The development of cox-1 and cox-2 inhibitors: A review. Int. J. Pharm. Sci. Res..

[4] Huynh N.C., Nguyen T.T.T., Nguyen D.T.C., Tran T.V. (2023). Occurrence, toxicity, impact and removal of selected non-steroidal anti-inflammatory drugs (NSAIDs): A review. Sci. Total Environ..

[5] Brennan R., Shawabkeh H., Wazaify M., Boardley I., McVeigh J., Van Hout M.C. (2021). A scoping review of non-medical and extra-medical use of non-steroidal anti-inflammatory drugs (NSAIDs). Drug Saf..

[6] Browne N., Conneely M., Hudson C. (2022). Use of Non-Steroidal Anti-Inflammatory Drugs and Attitudes to Pain in Pasture-Based Dairy Cows: A Comparative Study of Farmers and Veterinarians. Front. Vet. Sci..

[7] Tsekouras N., Christodoulopoulos M.A.B., Meletis E., Kousoulis C., Kostoulas P., Pantazis V., Papatsiros V.G., Dimoveli K., Gougoulis D. (2025). Associations Between Non-Steroidal and Steroidal Anti-Inflammatory Drug Use, Welfare, and Milk Production in Dairy Sheep: A Multivariate Study. Animals.

[8] Izadi P., Izadi P., Salem R., Papry S.A., Magdouli S., Pulicharla R., Brar S.K. (2020). Non-steroidal anti-inflammatory drugs in the environment: Where were we and how far we have come?. Environ. Pollut..

[9] Jurado A., Vázquez-Suñé E., Pujades E. (2021). Urban Groundwater Contamination by Non-Steroidal Anti-Inflammatory Drugs. Water.

[10] Aranda-Lara L., Gomez-Olivan L.M. (2020). Non-Steroidal Anti-Inflammatory Drugs in Water: Emerging Contaminants and Ecological Impact.

[11] Patel N., Khan M.D., Shahane S., Rai D., Chauhan D., Kant C., Chaudhary V.K. (2020). Emerging Pollutants in Aquatic Environment: Source, Effect, and Challenges in Biomonitoring and Bioremediation—A Review. Pollution.

[12] Wilkinson J., Hooda P.S., Barker J., Barton S., Swinden J. (2017). Occurrence, fate and transformation of emerging contaminants in water: An overarching review of the field. Environ. Pollut..

[13] Gale P.A., Caltagirone C. (2018). Fluorescent and colorimetric sensors for anionic species. Coord. Chem. Rev..

[14] Laxman W., Anushree T., Pawan K., Yong S., Samandhan P., Akash D., Ki H. (2017). Functionalized fluorescent nanomaterials for sensing pollutants in the environment: A critical review. Trends Anal. Chem..

[15] Das A.K. (2025). Advances in detecting non-steroidal anti-inflammatory drugs (NSAIDs) using molecular receptors and nanostructured assemblies. RSC Med. Chem..

[16] Chen S., Zhou S., Fu J., Tang S., Wu X., Zhao P., Zhang Z. (2021). A near infrared fluorescence imprinted sensor based on zinc oxide nanorods for rapid determination of ketoprofen. Anal. Methods.

[17] Delgado-Pérez T., Bouchet L.M., de la Guardia M., Galian R.E., Pérez-Prieto J. (2013). Sensing chiral drugs by using CdSe/ZnS nanoparticles capped with N-acetyl-L-cysteine methyl ester. Chem. Eur. J..

[18] Molina-García L., Santos J.L.M., Ruiz-Medina A., Llorent-Martínez E.J. (2013). Determination of ketoprofen based on its quenching effect in the fluorescence of quantum dots. J. Food Drug Anal..

[19] Bhogal S., Kaur K., Maheshwari S., Malik A.K. (2019). Surface Molecularly Imprinted Carbon Dots Based Core-Shell Material for Selective Fluorescence Sensing of Ketoprofen. J. Fluoresc..

[20] Wang T., Li Q., Wang M., Xu J., Li J., Wang F. (2023). Synthesis of fluorescent artificial receptors with high specificity for simultaneous detection of non-steroidal anti-inflammatory drugs. Food Chem..

[21] Mabrouk M., Hammad S.F., Abdella A.A., Mansour F.R. (2021). A novel strategy for ketorolac detection based on turn-on plasmonic enhanced FRET synchronous fluorometric sensor employing micellized chitosan/AgNPs nanocomposites: Preparation and mechanism investigation. Colloids Surf. A.

[22] Saini A., Kaur M., Mayank S., Kuwar A., Kaur N., Singh N. (2020). Hybrid nanoparticle based fluorescence switch for recognition of ketoprofen in aqueous media. Mol. Syst. Des. Eng..

[23] Jouyban A., Rahimpour E. (2020). Optical sensors based on silver nanoparticles for determination of pharmaceuticals: An overview of advances in the last decade. Talanta.

[24] Sun X., Liu P., Mancin F. (2018). Sensor arrays made by self-organized nanoreceptors for detection and discrimination of carboxylate drugs. Analyst.

[25] Liu Y., Minami T., Nishiyabu R., Wang Z., Anzenbacher P. (2013). Sensing of carboxylate drugs in urine by a supramolecular sensor array. J. Am. Chem. Soc..

[26] Akdeniz A., Mosca L., Minami T., Anzenbacher P. (2015). Sensing of enantiomeric excess in chiral carboxylic acids. Chem. Commun..

[27] Pushina M., Koutnik P., Nishiyabu R., Minami T., Savechenkov P., Anzenbacher P. (2018). Anion Sensing by Fluorescent Expanded Calixpyrroles. Chem. Eur. J..

[28] Akdeniz A., Minami T., Watanabe S., Yokoyama M., Ema T., Anzenbacher P. (2016). Determination of enantiomeric excess of carboxylates by fluorescent macrocyclic sensors. Chem. Sci..

[29] Picci G., Aragoni M.C., Arca M., Caltagirone C., Formica M., Fusi V., Giorgi L., Ingargiola F., Lippolis V., Macedi E. (2023). Fluorescent sensing of non-steroidal anti-inflammatory drugs naproxen and ketoprofen by dansylated squaramide-based receptors. Org. Biomol. Chem..

[30] Romano G.M., Mummolo L., Savastano M., Paoli P., Rossi P., Prodi L., Bencini A. (2022). Polyamine receptors containing anthracene as fluorescent probes for ketoprofen in H_2_O/EtOH solution. Chem. Commun..

[31] Paderni D., Macedi E., Giacomazzo G.E., Formica M., Giorgi L., Valtancoli B., Rossi P., Paoli P., Conti L., Fusi V. (2024). A new biphenol-dipicolylamine based ligand and its dinuclear Zn^2+^ complex as fluorescent sensors for ibuprofen and ketoprofen in aqueous solution. Dalton Trans..

[32] Martell E., Smith R.M., Motekaitis R.J. (2004). NIST Standard Reference Database 46. NIST Critically Selected Stability Constants of Metal Complexes Database, Version 8.0.

[33] Alarcón J., Aucejo R., Albelda M.T., Alves S., Clares M.P., García-España E., Lodeiro C., Marchin K.L., Parola A.J., Pina F. (2004). Fluorescent type II materials from naphthylmethyl polyamine precursor. Supramol. Chem..

[34] Valeur B. (2001). Molecular Fluorescence Principles and Applications.

[35] Alves S., Pina F., Albelda M., García-España E., Soriano C., Luis S.V. (2001). Open-Chain Polyamine Ligands Bearing an Anthracene Unit–Chemosensors for Logic Operations at the Molecular Level. Eur. J. Inorg. Chem..

[36] Bazzicalupi C., Bencini A., Bianchi A., Giorgi C., Fusi V., Valtancoli B., Bernardo M.A., Pina F. (1999). Effect of protonation and Zn(II) coordination on the fluorescence emission of a phenanthroline-containing macrocycle. An unusual case of “nonemissive” Zn(II) complex. Inorg. Chem..

[37] Gans P., Sabatini A., Vacca A. (1996). Investigation of equilibria in solution. Determination of equilibrium constants with the HYPERQUAD suite of programs. Talanta.

[38] Irving H., Williams R.J.P., Ferrett D.J., Williams A.E. (1954). The influence of ring size upon the stability of metal chelates. J. Chem. Soc..

[39] Cukrowski I., Cukrowska E., Hancock R.D., Anderegg G. (1995). The effect of chelate ring size on metal ion size-based selectivity in polyamine ligands containing pyridyl and saturated nitrogen donor groups. Anal. Chim. Acta.

[40] Lee B., Richards F.M. (1971). The interpretation of protein structures: Estimation of static accessibility. J. Mol. Biol..

[41] Mitternacht S. (2016). FreeSASA: An open-source C library for solvent accessible surface area calculations. F1000Research.

[42] Tan S.S., Kim S.J., Kool E.T. (2011). Differentiating between fluorescence-quenching metal ions with polyfluorophore sensors built on a DNA backbone. J. Am. Chem. Soc..

[43] Nugent J.W., Lee H., Lee H.S., Reibenspies J.H., Hancock R.D. (2014). The effect of π contacts between metal ions and fluorophores on the fluorescence of PET sensors: Implications for sensor design for cations and anions. Inorg. Chem..

[44] Romano G.M., Simonini Steiner Y.T., Bartoli F., Conti L., Macedi E., Bazzicalupi C., Rossi P., Paoli P., Innocenti M., Bencini A. (2025). Selective binding and fluorescence sensing of Zn(II)/Cd(II) using macrocyclic tetra-amines with different fluorophores: Insights into the design of selective chemosensors for transition metals. Dalton Trans..

[45] Bartoli F., Bencini A., Conti L., Giorgi C., Valtancoli B., Paoli P., Rossi P., Le Bris N., Tripier R. (2016). Catching anions with coloured assemblies: Binding of pH indicators by a giant-size polyammonium macrocycle for anion naked-eye recognition. Org. Biomol. Chem..

[46] Adams M.J., Highfield J.C., Kirkbright G.F. (1997). Determination of absolute fluorescence quantum efficiency of quinine bisulfate in aqueous medium by optoacoustic spectrometry. Anal. Chem..

[47] Hoover W.G. (1985). Canonical dynamics: Equilibrium phase-space distributions. Phys. Rev. A.

[48] Hoover W.G. (1986). Constant-pressure equations of motion. Phys. Rev. A.

[49] Parrinello M., Rahman A. (1981). Polymorphic transitions in single crystals: A new molecular dynamics method. J. Appl. Phys..

[50] Hornak V., Abel R., Okur A., Strockbine B., Roitberg A., Simmerling C. (2006). Comparison of multiple amber force fields and development of improved protein backbone parameters. Proteins.

[51] Bayly C.I., Cieplak P., Cornell W.D., Kollman P.A. (1993). A wellbehaved electrostatic potential based method using charge restraints for deriving atomic charges: The RESP model. J. Phys. Chem..

[52] Li P., Roberts B.P., Chakravorty D.K., Merz K.M. (2013). Rational Design of Particle Mesh Ewald Compatible Lennard-Jones. Parameters for +2 Metal Cations in Explicit Solvent. J. Chem. Theory Comput..

[53] Jorgensen W.L., Chandrasekhar J., Madura J.D., Impey R.W., Klein M.L. (1983). Comparison of simple potential functions for simulating liquid water. J. Chem. Phys..

[54] de Oliveira Cavalcante A., Chelli R. (2021). Polarizability relaxation in water/ethanol mixtures. J. Mol. Liq..

[55] Essmann U., Perera M.L., Berkowitz M.L., Darden T., Lee H., Pedersen G.L. (1995). A smooth particle mesh Ewald method. J. Chem. Phys..

[56] Tuckerman M.E., Berne B., Martyna G. (1992). Reversible multiple time scale molecular dynamics. J. Chem. Phys..

[57] Marsili S., Signorini G., Chelli R., Marchi M., Procacci P. (2010). ORAC: A molecular dynamics simulation program to explore free energy surfaces in biomolecular systems at the atomistic level. J. Comput. Chem..

[58] Becke A.D. (1988). Density-functional exchange-energy approximation with correct asymptotic behavior. Phys. Rev. A.

[59] Lee C., Yang W., Parr R.G. (1988). Development of the Colle-Salvetti correlation-energy formula into a functional of the electron density. Phys. Rev. B.

[60] Vosko H., Wilk L., Nusair M. (1980). Accurate spin-dependent electron liquid correlation energies for local spin density calculations: A critical analysis. Can. J. Phys..

[61] Grimme S., Ehrlich S., Goerigk L. (2011). Effect of the damping function in dispersion corrected density functional theory. J. Comp. Chem..

[62] Dunning T.H., Hay P.J., Schaefer H.F. (1977). Methods of electronic structure theory. Modern Theoretical Chemistry.

[63] Tomasi J., Mennucci B., Cammi R. (2005). Quantum mechanical continuum solvation models. Chem. Rev..

[64] Frisch M.J., Trucks G.W., Schlegel H.B., Scuseria G.E., Robb M.A., Cheeseman J.R., Scalmani G., Barone V., Petersson G.A., Nakatsuji H. (2016). Gaussian 16.

