# Supplementary material for: Ketoprofen Recognition and Sensing by Zn(II) Complexes with Fluorogenic Triamine Receptors
Source: Molecules. 2025 Nov 26;30(23):4556. doi: 10.3390/molecules30234556 (PMC12693136; doi:10.3390/molecules30234556)
Supplement: Supplementary file 1 [file molecules-30-04556-s001.zip › molecules-3962912-SI.pdf]

Ketoprofen recognition and sensing by Zn(II) complexes with fluorogenic triamine receptors

Yschtar "Tecla" Simonini Steiner [1], Liviana Mummolo [2], Rania Zartit [1], Massimo Innocenti [1], Marco Bonechi [1], Matteo Savastano [3], Luca Prodi [2*], Andrea Bencini [1*], Riccardo Chelli [1*], Giammarco Maria Romano [1]

[1] Dipartimento di Chimica "Ugo Schiff", Università degli Studi di Firenze, Via della Lastruccia 3, 50019-Sesto Fiorentino, Firenze, Italy.
[2] Dipartimento di Chimica "Giacomo Ciamician", Università degli Studi di Bologna, Via Gobetti 85, 40129 Bologna, Italy.
[3] Dipartimento di Scienze Umane e Promozione della Qualità della Vita, Università San Raffaele Roma, Via di Val Cannuta 247, 00166 Rome, Italy.
* Correspondence: luca.prodi@unibo.it (L.P.); andrea.bencini@unifi.it (A.B.); riccardo.chelli@unifi.it (R.C.)

Supplementary Materials

Table S1. Average solvent accessible surface area, SASA, (Å^2) of the anthracene units of the systems under study. For each system, the SASA has been computed averaging over the time and over the two anthracene moieties.

	L ≡ L1	L ≡ L2
L	285	264
$[ZnL]^{2+}$	308	257
$KP[ZnL]^{+}$	260	249
$KP_2[ZnL]$	209	230

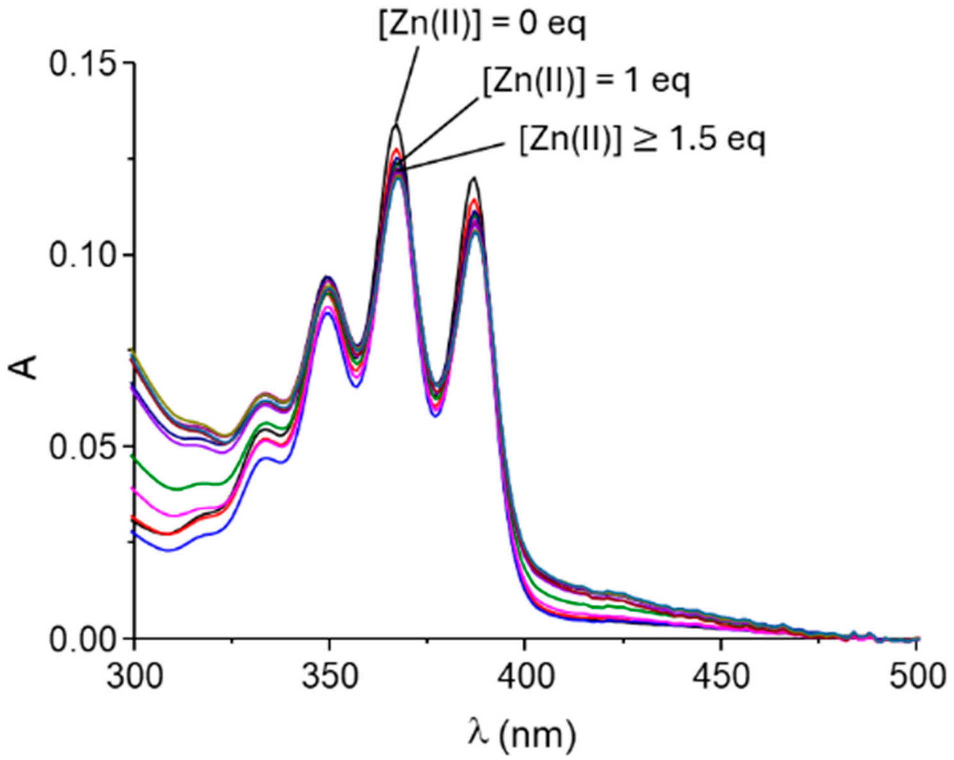

Figure S1. Absorption spectra of L2 in the presence of increasing amount of Zn(II) at neutral pH values ([L2] = 1×10^{-5} M, TRIS/HCl buffer 0.001 M, λ_{exc} = 340 nm, 298 K).

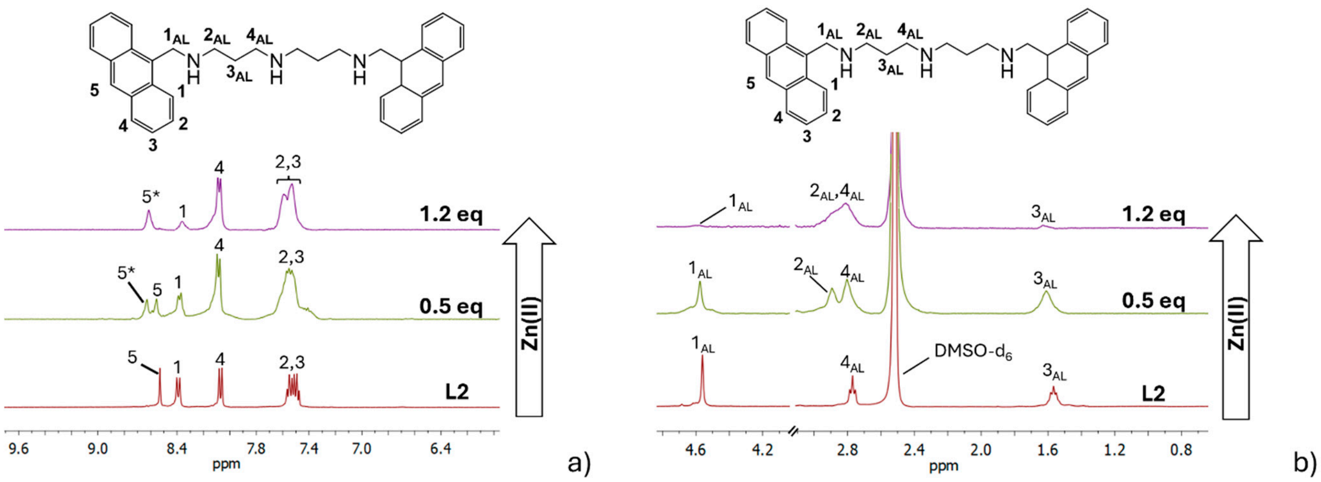

Figure S2. Aliphatic (a) and aromatic (b) portions of the ^{1}H NMR spectrum of L2 in the absence and in the presence of 0.5 and 1 equivalent of Zn(II) in DMSO at 298 K ([L1] = 0.01 M). Starred numbers indicate the ^{1}H signals in the Zn(II) complex.

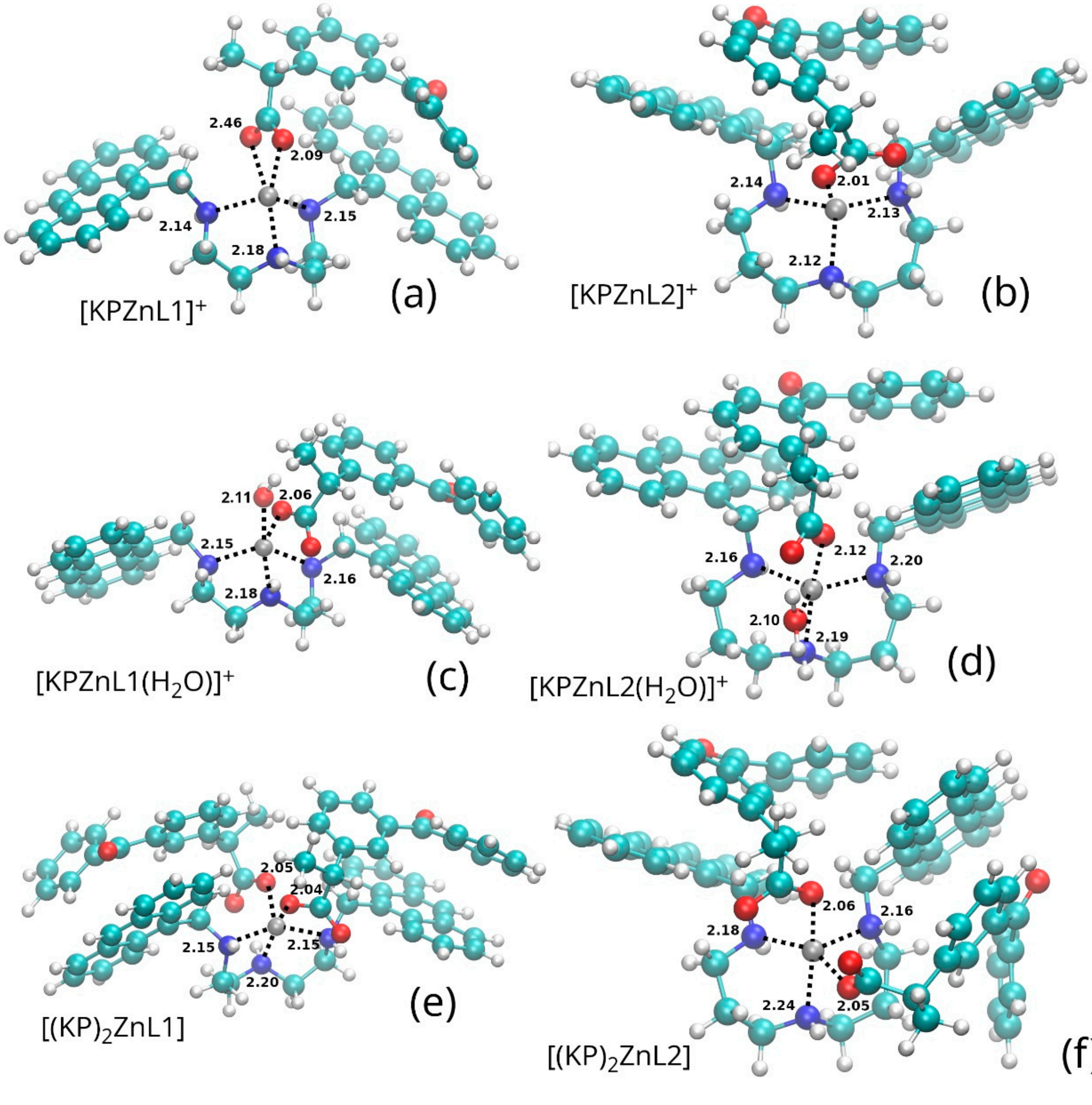

Figure S3. Ball and stick representations of energetically optimized structures of the $[KPZnL]^+$ (a, b), $[KPZnL(H_2O)]^+$ (c, d) and $[(KP)_2ZnL]$ (e, f) complexes (with L = L1 or L2), obtained from ab initio calculations. Cyan: C, blue: N, red: O, white: H, grey: Zn. The Zn-N and Zn-O(KP) bond distances (Å) are also shown.
